# Kinetic Exclusion Assay of Biomolecules by Aptamer Capture

**DOI:** 10.3390/s20123442

**Published:** 2020-06-18

**Authors:** Mark H. Smith, Daniel Fologea

**Affiliations:** 1 Department of Physics, Boise State University, 1910 University Drive, Boise, ID 83725, USA; marksmith12@u.boisestate.edu; 2 Biomolecular Sciences Graduate Programs, Boise State University, 1910 University Drive, Boise, ID 83725, USA

**Keywords:** KinExA, aptamer, thrombin, affinity, concentration determination

## Abstract

DNA aptamers are short nucleotide oligomers selected to bind a target ligand with affinity and specificity rivaling that of antibodies. These remarkable features recommend aptamers as candidates for analytical and therapeutic applications that traditionally use antibodies as biorecognition elements. Numerous traditional and emerging analytical techniques have been proposed and successfully implemented to utilize aptamers for sensing purposes. In this work, we exploited the analytical capabilities offered by the kinetic exclusion assay technology to measure the affinity of fluorescent aptamers for their thrombin target and quantify the concentration of analyte in solution. Standard binding curves constructed by using equilibrated mixtures of aptamers titrated with thrombin were fitted with a 1:1 binding model and provided an effective K_d_ of the binding in the sub-nanomolar range. However, our experimental results suggest that this simple model does not satisfactorily describe the binding process; therefore, the possibility that the aptamer is composed of a mixture of two or more distinct K_d_ populations is discussed. The same standard curves, together with a four-parameter logistic equation, were used to determine “unknown” concentrations of thrombin in mock samples. The ability to identify and characterize complex binding stoichiometry, together with the determination of target analyte concentrations in the pM–nM range, supports the adoption of this technology for kinetics, equilibrium, and analytical purposes by employing aptamers as biorecognition elements.

## 1. Introduction

Aptamers are short nucleotide oligomers selected and isolated by the Systematic Evolution of Ligands by Exponential Enrichment (SELEX) technique [1,2,3,4] to present high specificity and affinity for a large variety of targets ranging from ions [5,6,7,8] and small molecules [9,10,11,12] to whole cells [13,14,15]. Their ability to selectively recognize and bind targets recommends them as promising alternatives to antibodies for scientific and medical purposes [9,13,16,17,18,19,20,21,22,23,24,25]. Aptamers have been investigated as potential analytical and diagnostic tools with approaches and technologies that traditionally employ antibodies as recognition elements. Compared to antibodies, DNA aptamers are cost effective, present similar specificity and affinity for targets, have greater stability either lyophilized or in solution at room temperature, and are readily amenable to chemical modifications [17,25,26,27,28].

Numerous traditional and emerging analytical techniques have been proposed for aptamer-based qualitative and quantitative assessments of molecular and cellular interactions [23,25,28,29,30,31,32]. Aptamer-thrombin systems that use the 15-mer [33] and/or 29-mer [34] aptamer as biorecognition elements are among the most used in scientific investigations focused on affinity and concentration determination [29,35,36,37,38,39,40,41], which is justified by the essential physiological role of thrombin [42]. Nonetheless, both aptamers are far from fully characterized in terms of affinity and binding models [35]. For example, the reported affinity of the 29-mer aptamer varies by more than four orders of magnitude, and is also strongly dependent on the instrumental method and adopted binding model [29,34,35,36,37,39,40,41]. Only a few studies reported affinities in the nM range [40,41], close to the ~0.5 nM first reported by Tasset [34], while many others indicated affinities up to a few hundred times larger [29,35,36,37,39]. It is not clear if such discrepancies originate in the instrumentation and mathematical models employed, or whether particular experimental conditions and methodologies also influence the measurements [35]. To better understand the aptamer-thrombin interactions and pave the way towards analytical applications, we used the kinetics exclusion assay (KinExA) technology, developed by Sapidyne, Inc. (Boise, ID, USA) to determine the 29-mer aptamer affinity for thrombin, and thrombin concentration in solutions.

This technology provides highly accurate analyses of molecular interactions in true solution phase systems [43,44,45,46,47,48,49,50,51,52,53,54]. The principle of operation relies on specifically assessing only the unbound partner in a mixture of bound and unbound molecules [55,56,57,58]. The KinExA instrument is an automatic solution-handling system equipped with a sensitive and versatile fluorescence detection system [55,59,60,61]. To perform measurements, a small volume of a bi-molecular reaction mixture is flowed into a low-volume flow cell over a solid phase consisting of small beads functionalized to specifically bind one of the partners [52,56,62]. The contact time between the beads and solution is brief enough that any dissociation of the bi-molecular complex is insignificant. The beads specifically capture a small fraction of the free partner, proportional to total free molecule in solution, which is quantified by either intrinsic fluorescence of the captured molecules or by using fluorescent secondary probes (i.e., specific labels, anti-tag, or anti-species secondary antibody) that do not participate in the primary interaction in the solution phase [63,64]. Depending on the experiment goals, the resulting fluorescence signal measured directly on the beads is analyzed using various binding models and protocols to determine kinetics, affinity, or concentration [48,50,55,58,65,66,67,68,69].

Although this technology has been chiefly used with antibodies as biorecognition elements [46,54,55,56,70], the only restriction on recognition molecules is that at least one of them needs to be fluorescently detectable, either directly or indirectly [55]. We used this technology to interrogate an aptamer-thrombin binding system, and our protocols make full use of the advantages offered by the KinExA technology in terms of automation, accuracy, reliability, and adaptability. In addition, the use of aptamers enables using a complementary DNA on a solid phase [71,72,73,74] to specifically capture the free aptamer in solution (unbound to the target thrombin). By introducing a fluorescently-labeled aptamer as a specific biorecognition element for thrombin, the detection of the unbound aptamer can be achieved without using a secondary label. Our results demonstrate that the KinExA platform is directly applicable to aptamers for determining their affinity for target, whether simple or complex binding stoichiometries are considered, and for measuring concentration of target molecules.

## 2. Materials and Methods

The 29 nucleotide thrombin-specific aptamer (sequence 5′-AGTCCGTGGTAGGGCAGGTTGGGGTGACT-3′), identified by Tasset et al. [34] was obtained by custom order from Integrated DNA Technologies, Inc. (IDT, Coralville, IA, USA). At our request, the supplier provided the aptamer modified at the 5′ end by the addition of the fluorophore Alexa Fluor 647 to enable direct fluorescent detection. For the capture DNA (cDNA) strand, we selected a 16-nucleotide sequence complementary to bases 11–26, with two additional thymine residues and biotin at the 3′ end (5′-CACCCCAACCTGCCCTTT-biotin-3′). This material was also obtained by custom order from IDT. The DNA strands were reconstituted with nuclease-free water to a stock concentration of 100 µM. Sample solutions consisting of fluorescent aptamer (FA) with various concentrations of thrombin (MilliporeSigma, St. Louis, MO, USA) being prepared in sample buffer (SB), consisting of phosphate buffered saline with 0.02% sodium azide (PBS, Sapidyne Instruments) supplemented with 1 mg/mL bovine serum albumin (BSA, Sapidyne Instruments). The running buffer consisted of PBS alone.

### 2.1. Solid Phase and Sample Preparation, and Data Collection with the KinExA 3200 Instrument 

**Figure 1:**
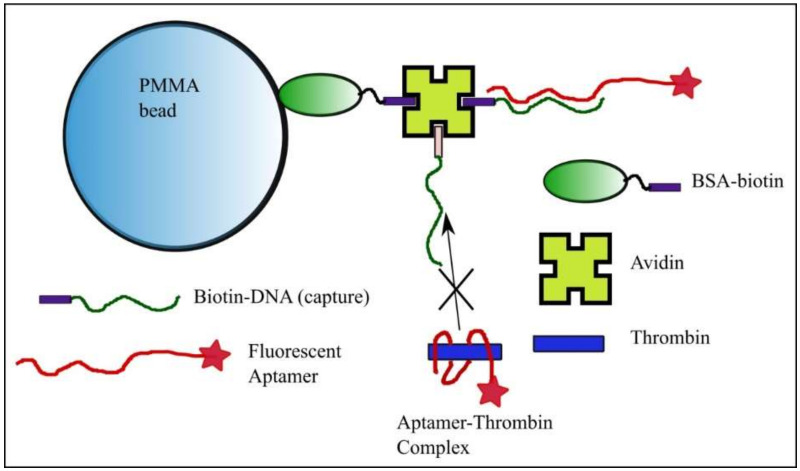
Bead preparation for equilibrium and concentration measurements with the kinetics exclusion assay technology. Polymethylmethacrylate (PMMA) beads functionalized with bovine serum albumin (BSA)-biotin and avidin serve as anchoring elements for the DNA strand with sequence complementary to a portion of the fluorescent aptamer. The free aptamer is able to bind the complementary strand anchored to the bead, while binding of the aptamer-thrombin complex is prevented.

The multi-step preparation of functionalized beads as solid phase for capturing the free FA in solution is shown schematically in Figure 1. Following the manufacturer’s guidance, 1 mL of 20 µg/mL BSA-biotin (MilliporeSigma) in PBS was mixed with 200 mg of polymethylmethacrylate (PMMA) beads of 98 µm average diameter (Sapidyne Instruments). The beads were rotated for two hours at room temperature, after which we performed five steps of gravity pelleting/PBS washing. Next, the beads were exposed to 100 µg/mL egg white avidin (MilliporeSigma) in PBS containing 10 mg/mL BSA and rotated for two hours to functionalize them for anchoring the biotinylated cDNA molecule through the strong biotin-avidin bond. After five steps of gravity pelleting/PBS washing, one vial of beads functionalized with BSA-biotin and avidin (BSA-B-A) was reserved for preliminary control experiments, while another vial underwent a last step of functionalization by immobilizing the biotinylated cDNA on the beads, following similar equilibration and washing procedures in which we used as bathing solution 1 mL of 2 µM biotinylated cDNA in PBS. The cDNA functionalized beads (BSA-B-A-cDNA) were further used for control, equilibrium, and concentration measurement experiments.

**Figure A1:**
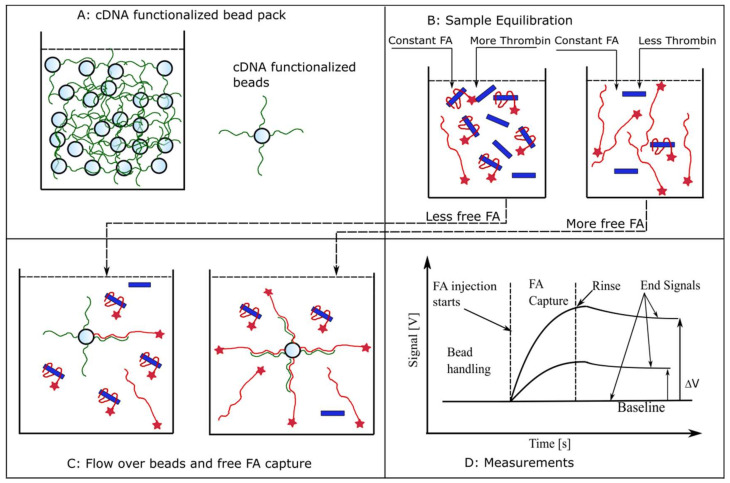
Using aptamers as biorecognition elements for kinetic exclusion assay measurements. (**A**) A pack of beads functionalized with cDNA for FA capture is introduced in the flow cell. (**B**) Samples consisting of constant FA concentrations and variable thrombin amounts are equilibrated in test tubes. (**C**) Aliquots of equilibrated samples are flowed over the beads in the flow cell and a fraction of free FA is captured. This fraction is inversely proportional to the amount of thrombin added during the equilibration step. (**D**) The expected evolution of the fluorescence signal in response to different concentrations of free FA in solution. FA injection produces an increasing fluorescence signal owing to its capture and accumulation onto beads. The end signal recorded after the unbound FA is rinsed from the flow cell is proportional to the amount of free FA present in the equilibrated sample (not complexed with thrombin). For identical label (FA) concentrations in equilibrated solutions, the end signal decreases as the concentration of the target molecule (thrombin) increases, indicative of a reduced availability of the free, unbound label.

For the control experiments, we utilized the BSA-B-A and BSA-B-A-cDNA functionalized beads, which were automatically injected into the flow cell of the KinExA 3200 instrument with autosampler (Sapidyne Instruments). The controls included samples consisting of PBS alone (for baseline) or 50 pM FA prepared in SB. The equilibrium measurements (see Figure A1 and the explanations provided in Appendix A) employed a constant concentration of FA (i.e., 10 pM, 50 pM, and 10 nM) in SB, mixed with thrombin at final concentrations up to 1 µM, and equilibrated for at least two hours at room temperature. To demonstrate the potential of the technology for concentration and diagnostic testing using aptamers, we prepared mixtures of FA (50 pM) and thrombin (5 nM, 2.5 nM, and 0.5 nM). These samples were equilibrated at room temperature for two hours.

Aliquots of samples were passed over the functionalized beads in the flow cell of the instrument. A fresh aliquot of functionalized beads was used for each sample and replaced automatically using the default protocol supplied with the instrument. Sample measurement steps consisted of initiation with a flow of 100 µL of PBS, injection of 1 mL of the analytical sample (or control), and injection of 1 mL of PBS, all at a flow rate of 0.5 mL/min. This was followed by a final rinse with 1.5 mL of PBS at a rate of 1.5 mL/min. The fluorescence data from the detector (expressed in volts) was recorded at a rate of one sample/second during the above steps and plotted for data interpretation (see Figure A1 in Appendix A for reference) and further analysis. The same measurement steps were followed throughout for concentration measurements. 

### 2.2. Data Analysis

Details of the signal equation used for fitting the 1:1 binding model [55,59,75,76], n-curve analysis module [54,67,70,76], mixed model [75,77], and logistic equation for concentration determination [61,78] are provided in Appendix A. The data were analyzed and plotted with KinExA Pro software (version 4.4.36, Sapidyne Instruments), and Origin 8.5.1 software (Origin Labs, Northampton, MA, USA).

## 3. Results and Discussion

In this work, we investigated the suitability of the kinetic exclusion assay technology for measuring an aptamer’s binding affinity for its designated target, and for using the aptamer as a recognition element in a bioassay for thrombin concentration measurements. Our proposed investigative platform employs BSA-B-A-cDNA functionalized beads to capture a fraction of the free FA remaining in equilibrated mixtures of analyte (thrombin) and FA. A potential impediment to this approach would be an unexpected strong, non-specific binding of the aptamer molecules to functionalized beads in the absence of cDNA immobilized on their surface. To address this issue, we performed preliminary investigations of specific and non-specific binding by employing functionalized beads without and with cDNA immobilized on their surface (control and capture beads, respectively) and running the experiments with 1 mL of either FA-free SB or SB containing 50 pM FA. 

**Figure 2:**
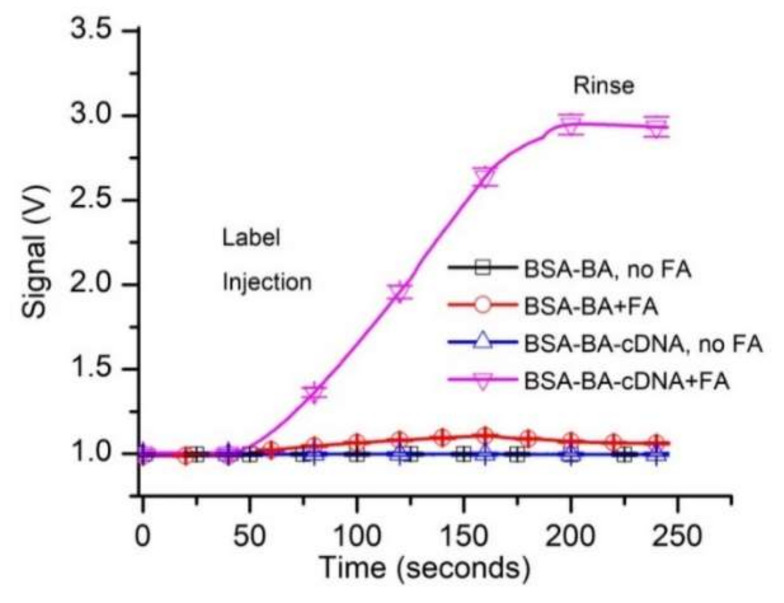
Preliminary testing of the fluorescent aptamer’s suitability for equilibrium and concentration measurements by kinetic exclusion assay. The tests investigated specific and non-specific binding of 50 pM fluorescent aptamer (FA) to control and capture beads. All the data in the graphs represent experimental values, with the symbols added for identification. Each curve shows the average values of experimental data (n = 3, ±SD).

The analysis of the evolution of the raw fluorescence signal (Figure 2) shows steady, almost identical baselines (~1 V absolute signal value) recorded for both types of beads in the absence of FA in the solutions. When the FA sample reached the flow cell containing control beads, the fluorescence started to slowly increase and reached a maximum value of ~1.1 V (~0.1 V above baseline). Such a small change of the signal was expected since the FA concentration in the flow solution was very low (50 pM). During the rinsing step, the free FA was washed away with the buffer, as inferred from the gradual decrease of the fluorescence signal. The small difference from the baseline recorded at the end of the trace versus baseline indicated a weak, non-specific binding (NSB).

When the FA-containing sample was flowed over capture beads (BSA-B-A-cDNA), the fluorescence signal increased significantly (Figure 2) and reached an absolute value of ~2.9 V (~1.9 V above baseline). This much larger signal may be explained by a continuous accumulation of the FA in the flow cell as it was captured by the cDNA immobilized on the capture beads. The signal decreased by only a small fraction during the rinsing process, suggesting that the FA bound very strongly to the capturing beads. This was anticipated since the resulting duplex DNA, although relatively short, has a predicted melting temperature of 58.4 °C and the estimated free energy for hybridization is large (∆G = −39 kcal/mole; supplier data).

The specific binding of free FA resulted in a strong increase in the fluorescence signal (~1.9 V above the baseline), while the non-specific binding led to a change in the signal of less than 0.1 V. Consequently, we concluded that the experimental system was adequate for initiating further investigation of affinity and concentration measurements.

**Figure 3:**
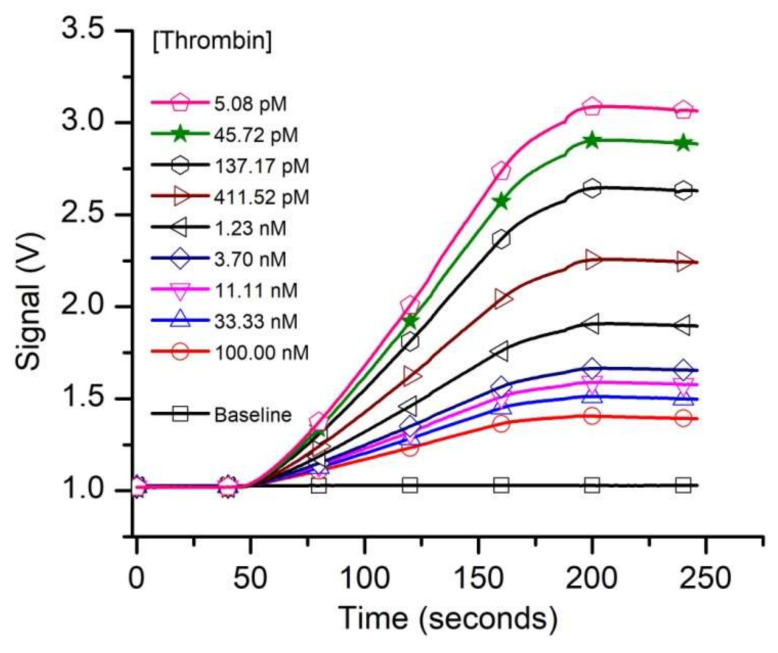
The evolution of the raw fluorescence signal recorded from capturing the free FA left in solutions equilibrated with various thrombin concentrations. The free FA availability decreased as the titrant concentration in the samples increased. For this experiment we utilized 50 pM FA as constant binding partner (CBP).

Having established the existence of specific FA binding to our functionalized solid phase, we turned our attention to determining the affinity of the aptamer for thrombin from kinetic exclusion assay experiments. We assumed that the FA-thrombin complex will not be able to form a duplex with the cDNA; therefore, specific capture of the complex will be prevented. In accordance with standard practice for n-curve analysis, we set up three experimental curves, for which we adjusted the concentration of the FA in SB (i.e., 10 pM, 50 pM, and 1 nM, respectively), used as a constant binding partner (CBP) for each curve. The thrombin amount in the sample tubes was adjusted (titrated) for final concentrations ranging from 5 pM to 1 µM, and we used up to 18 thrombin concentrations (including no thrombin samples) for the experiments. The 1 nM and 50 pM FA samples were incubated for two hours, and the 10 pM FA samples were incubated for 6.5 h before measurements with the KinExA 3200 instrument, and each sample set was run at least in duplicate. A typical run for the sample set that utilized 50 pM FA as CBP is shown in Figure 3. The plot clearly indicates that the fluorescence signal, indicative of free FA in the equilibrated solutions decreased significantly as the titrant (thrombin) concentration increased. The slope of the traces increased with the amount of free FA in the equilibrated samples (therefore, it decreased as more thrombin was added to the reaction tubes), and confirmed the signal rate’s dependency on the concentration of binding partner [74].

**Figure 4:**
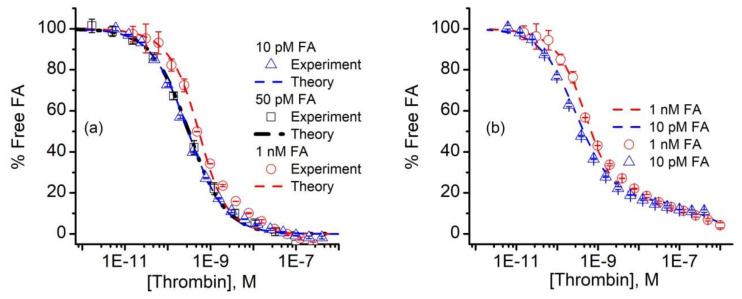
Analysis of FA binding data. (**a**) The n-curve analysis provided the calculated percent free FA (symbols) and theoretical simulations (dashed lines) for 10 pM, 50 pM, and 1 nM FA in the thrombin-titrated solutions for a 1:1 binding model. (**b**) The 10 pM and 1 nM FA signal data (symbols) were fitted with a binding model (dashed lines) that comprised a hypothetical mixture of two competing aptamers, characterized by different K_d_ values. The experimental data represents average values ± SD (n = 3 for 50 pM FA, and n = 2 for 10 pM and 1 nM FA).

The curves corresponding to the three different FA concentrations were simultaneously analyzed with the n-curve analysis module [54,67,70,76] included with the KinExA Pro software package to determine the equilibrium dissociation constant K_d_. The calculated percent of free FA vs. thrombin concentration and the theoretical curves for the three FA concentrations for a 1:1 binding model (Equation (A1)) are shown in Figure 4a.

The n-curve analysis provided a K_d_ of 298 pM (+111/−81 pM), which is in good agreement with previous estimates of the same aptamer-thrombin system of ~0.5 nM [34], or a few nM [40,41]. We also noted that the 10 pM and 50 pM FA curves were nearly identical (Figure 4a), which can only occur when the thrombin concentration corresponding to the 50% inhibition point (approximately 300 pM) of both curves is equal to the K_d_ [59]. In addition to data overlapping, the 10 pM and 50 pM FA concentrations exhibited a better fit than the 10 nM FA samples. Even so, one may observe a distinct deviation of the data from the 1:1 binding model used for analysis (i.e., Equation (A1)) for the higher thrombin concentrations. All the additional curves we obtained and analyzed showed the same systematic error in the same range. Aware that mixtures of antibodies can generate standard binding curves of similar appearance [75,77], we wondered if the fit discrepancies in Figure 4a could be the result of competition between two or more populations of FA, each with its own affinity for thrombin. To examine this hypothesis, we followed the example provided by Glass et al. [77] and set up a model that accounted for the competition of two FA of differing K_d_ for thrombin [59,75]. With this competitive binding model, we obtained a qualitatively better fit to the measured data (Figure 4b), which corresponded to a hypothetical two component FA mixture, in which 84% has a calculated K_d_ for thrombin of 298 pM, and the remaining 16% has a K_d_ of 500 nM. 

A better fit is not sufficient to prove that the dual binding model is the correct one, and we have no reason to believe that the mixture, if it exists, would have only two components. In this regard, we note that the fitted FA activity in Figure 4 was only ~47% for all FA concentrations, suggesting that half of the FA was actually inactive with regard to binding thrombin. Previous investigations on the same aptamer considered either a 1:1 binding model or models that imply multiple binding sites, including cooperativity and induced fit mechanisms [29,35,36,37,38,39,40,41]. Nonetheless, almost all of those approaches report significantly lower affinities, and the possibility of a mixed population was not addressed. The fluorescent label that we added to the aptamer may alter the binding and equilibrium, but most of the studies cited herein as comparison have also used labels or spacers as modifiers. We do not believe that this particular label addition led to such a significant increase in affinity, although this potential effect is worthy of further investigations. The complex binding observed in our case may be inherent to this aptamer, or may originate in a potential duplex formation between a shorter segment of the cDNA and a complementary sequence of the FA outside the thrombin binding site. Either way, it is a strength of the KinExA technology that deviation from 1:1 binding can be discerned in the collected data, which we anticipate will prompt further inquiry into mechanisms that may explain the observed binding curves for this and other aptamers.

**Figure 5:**
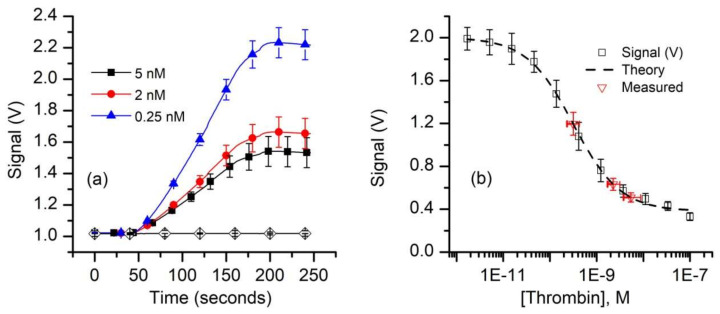
Equilibrium measurements of thrombin concentration by kinetic exclusion assay. (**a**) The raw fluorescence signal recorded for thrombin concentrations of 0.25 nM, 2 nM, and 5 nM (n = 3, ± SD) in equilibrated mixtures at a constant FA concentration of 50 pM. The symbols were added to facilitate curve discrimination and identification. (**b**) The end signal values measured against the baseline for standard and mock samples (symbols) were plotted and fitted (dashed line) with the four-parameter logistic equation (Equation (A2)) to determine the unknowns. The range of concentrations represented by the x error bars on the “measured” data points represent concentrations corresponding to the average measured signal (n = 3) ±SD of the measured signals.

Our next experiments aimed at evaluating the FA as a bioassay recognition element using kinetics exclusion assay technology. This task does not require a well-defined K_d_, only that we have a reproducible standard curve. To measure thrombin concentration by employing kinetic exclusion assay using aptamers as ligands, we included measurements of mock unknowns (i.e., 5.0 nM, 2.0 nM, and 0.25 nM thrombin concentration) in the 50 pM FA assay. Similar to the experimental results plotted in Figure 3, the resulting raw signal curves (Figure 5a) recorded with the equilibrated mock samples show that the end signal decreased substantially when the thrombin concentration increased, indicating reduced availability of FA in the equilibrated mixtures. 

Since the 1:1 binding model (Equation (A1)) proved unsatisfactory to best describe the experimental data, we used the four-parameter logistic (Hill slope) equation (Equation (A2)) [61,78] to fit the signal data for the standard curve (Figure 5b). The best fit parameter values calculated with this model are: upper asymptotic value B_1_ = 2.0 V, lower asymptotic plateau (NSB) B_2_ = 0.38 V, inflection point B_3_ = 0.32 nM, and a Hill slope B_4_ = 0.87. These parameters were also used to compute the “unknown” concentrations from the average values of the respective signals using Equation (A3). Our calculations provided average concentration values equal to 5.4 nM for the 5 nM sample, 2.2 nM for the 2.0 nM sample, and 0.32 nM for the 0.25 nM sample (Figure 5b). It is interesting that although only one of the samples (i.e., 0.25 nM thrombin) had a concentration near the middle of the curve, while the more concentrated samples were intentionally outside this region, the determined average concentration values were satisfactory. As expected, the uncertainty in the determined concentration is larger and asymmetrical for the higher concentrations because of the more gradual slope and curvature of the standard curve at these concentrations. From the signal curve (Figure 5b) one may easily observe that for this FA concentration, visible changes in the binding signal occurs for thrombin concentrations in the pM range; further optimizations of the FA concentration in relation to affinity may provide improved sensitivity, even in the sub-pM range [59].

## 4. Conclusions

This work strongly supports the hypothesis that the kinetic exclusion assay technology is suitable for kinetics and equilibrium measurements employing aptamers as specific biorecognition elements. Our experimental results demonstrate that both aptamer characterization and analyte concentration measurements are achievable through this methodology. All the advantages presented by this technology are anticipated to be maintained when aptamers are used as ligands for analytes. The KinExA technology does not suffer from surface matrix affinity effects [45,79], mass transport limitations, or mobility effects [55,80], does not require radioactive labels, avoids any surface-immobilization procedure that may alter binding constants compared to those measured in true solution phase, is highly accurate for repeated readings [81], and has no molecular weight limitations [55,82,83]. Multiple studies have revealed its superiority to SPR or ELISA in terms of accuracy, sensitivity, versatility, and reliability [45,48,49,76,81,82,84]. The instrument may assess, in various formats, the binding of partners ranging from ions [82,83] and small molecules [50,85,86] to large structures (e.g., whole cells, including fixed cells) [49,76,85,87], which makes aptamers an excellent choice for numerous kinetics, equilibrium, and concentration measurements by using the KinExA technology. The ability to identify complex binding stoichiometries may provide valuable insights into fundamental analyses of aptamer–analyte interactions, while its high sensitivity and accuracy recommend this technique for numerous bioanalytical applications.

## Appendix A

### Kinetics Exclusion Assay Principles

The principles of measurement and the anticipated evolution of the fluorescence signals during the various steps of the adapted protocol are briefly presented in Figure A1. The instrument automatically handles the packing of beads and the injection of all the required solutions into the microfluidic system, while monitoring the fluorescence signal of the label captured by the beads. In our case, the beads (Figure A1, panel A) are functionalized for capturing the free FA by following the procedures presented in Materials and Methods and Figure 1, and introduced into the flow cell. The reactants (FA and thrombin) are equilibrated in reaction tubes in which we kept constant the concentration of FA and varied the amount of thrombin (Figure A1, panel B). The concentration of free FA in pre-equilibrated solutions is in inverse proportion to the amount of thrombin added to the reaction tube. A fraction of the free FA is captured by the functionalized beads upon injection into the flow cell (Figure A1, panel C). For otherwise identical settings, the amount of captured FA is proportional to the amount of free FA; more thrombin in the equilibrated sample leads to less FA available for capture. FA injection after bead handling leads to an immediate increase in fluorescence signal owing to specific capture on the beads (Figure A1, panel D). The subsequent rinsing step washes away any non-captured FA, with the end signal representing only captured FA. This end signal is proportional to the amount of free FA that was injected in the previous step. It is assumed that the FA-thrombin complex (bound FA) will not bind to the cDNA, and the rate of dissociation of the pre-equilibrated complex is slow enough to be negligible during the brief residence time in the flow cell. Consequently, the end signal represents only binding of a fraction of FA free in equilibrated FA-thrombin solutions. An affinity value may be derived from further analysis of the relative changes in the end signal versus baseline (Figure A1, panel D) of a series of equilibrated solutions [55,59].

### Signal Equations and Modeling

The instrument end signal as a function of concentration for a 1:1 binding model is described by Equation (A1) [55,59,75,76]:

(A1) $$Signal=\frac{Sig_{100\%}-NSB}{2{\left[FA\right]}_{0}}\left(\left({\left[FA\right]}_{0}-K_{d}-{\left[T\right]}_{0}\right)+\sqrt{{\left[FA\right]}_{0}^{2}+2{\left[FA\right]}_{0}K_{d}+K_{d}^{2}+2{\left[T\right]}_{0}K_{d}+{\left[A\right]}_{0}^{2}}\right)+NSB$$

where *Sig*_100%_ represents the end signal recorded in the absence of thrombin in the mixture, *NSB* is the end signal corresponding to non-specific binding (or the signal when all of the aptamer is complexed with thrombin), and [*FA*]_0_ and [*T*]_0_ are the initial concentrations of *FA* and thrombin in the mixtures. This equation is fitted by the method of least squares to the experimental data obtained by using *FA* as CPB and thrombin as titrant. The n-curve analysis simultaneously fits Equation (A1) to multiple signal curves generated by using different CBP concentrations [54,67,70,76].

For the two-population aptamer mixture, the total concentration of *FA*, [*FA*]_0_, is assumed to be composed of two fractions [*FA*]_1_ = *f* × [*FA*]_0_, and [*FA*]_2_ = (1 − *f*) × [*FA*]_0_, where *f* is the fraction of the tightest binder in the population. We assumed that an *FA* molecule will provide the same signal irrespective of the population to which it belongs. For this fit, we used the 298 pM reported in the KinExA n-curve analysis as the tighter *K*_d_, and varied both the fraction *f* of the tightest binder and the *K*_d_ of the second fraction. The competitive equilibrium concentrations of *FA*_1_ and *FA*_2_ were calculated using a model described elsewhere [75,77]. We scaled the resulting combined free fraction of *FA* using a maximum binding signal (*Sig*_100%_) and offset it using a non-specific binding variable (NSB), as has been reported previously [59,75].

For the mock sample concentration measurement, the standard binding curve was obtained by averaging and fitting with a four-parameter logistic equation [61,78]:

(A2) $$Signal=\frac{B_{1}-B_{2}}{1+{\left(\frac{x}{B_{3}}\right)}^{B_{4}}}+B_{2}$$

where *B*_1_ is the upper asymptotic plateau (maximum signal), *B*_2_ is the lower asymptotic plateau (NSB), *B*_3_ is the inflection point (the concentration at which the signal is halfway between the extreme values), *B*_4_ is the Hill coefficient (slope), and *x* is the titrant’s concentration.

The same equation, rearranged to solve for *x*, allows calculation of the unknown concentrations from the corresponding signals:

(A3) $$\left[x\right]=\exp\left[\frac{\ln\left[\frac{-\left(-Signal+B_{1}\right)}{\left(-Signal+B_{2}\right)}\right]}{B_{4}}\right]\cdot B_{3}$$
